# Numerical computation of electrical potential on a gas evolving electrode

**DOI:** 10.1038/s41598-023-28592-2

**Published:** 2023-01-31

**Authors:** Soufiane Abdelghani-Idrissi, Annie Colin

**Affiliations:** MIE - Chemistry, Biology and Innovation (CBI) UMR8231, ESPCI Paris, CNRS, PSL Research University, 10 Rue Vauquelin, Paris, France

**Keywords:** Batteries, Hydrogen fuel

## Abstract

Electrochemical systems using a gas evolving electrode, such as metal-air batteries or electrolyzers, are confronted with recurrent problems related to gas production. Indeed, the production of gas at the surface of the electrodes causes a masking of the active surface which induces overvoltages and unstable electrical signals in time. We propose here numerical computations that take into account the spatial heterogeneity of the electrode and allow to account for the size distribution of the produced bubbles. We compare these computations to experiments on a Platinum–Carbon plate cell in the presence or absence of electrolyte flow. They reproduce the observed behavior and allow us to predict the stability of the signals. They are also a guide for the synthesis of efficient electrodes.

## Introduction

Our planet is a world in transition: demographic transition, climate transition, energy transition. The IPCC report, the recent heat waves in Canada, Europa, the floods in Europe, China in Pakistan remind us more and more that it is necessary to act for the climate. In this context, finding solutions to produce clean and renewable energy is the challenge of the 21st century. The main weakness of solar and wind energy is their intermittency. The electricity produced by these energies must therefore be stored. The development of sustainable and competitive batteries for the electrochemical energy storage is one of the crucial point for the large-scale deployment of renewable energy technologies^1^. Improving the batteries performances should make electric transport of people and goods competitive with thermal vehicles^2,^^3^. This context has recently given rise to significant research activity around metal-air batteries^4,^^5^. As examples, zinc-air and iron-air batteries start to be developed at an industrial scale in different countries, while these technologies are still improved by solving fundamentals issues^6^. These batteries are based on oxygen evolution and reduction reaction. During the discharge, the oxygen in the atmosphere is reduced thanks to a gas-diffusion electrode which plays the role of the anode and the metal is oxidised at the cathode. These processes are reversed during the charge phase : metallic hydroxide ions are reduced to solid metal electrodeposited at the electrode surface, while oxygen bubbles are formed by the oxidation of hydroxide ions at the opposite electrode. The main challenges to make these systems industrially viable are associated to the charge reactions^7,^^8^. At the metallic electrode, dendritic growth due to heterogeneous electrodeposition leads to a short-circuit inside the cell and thus governs the life-time of these systems. At the gas-evolving electrode, the production of bubbles prevents the possibility of fast charge cycles using high currents. It limits the total active area in contact with the electrolyte. This diminution of the active surface prevents locally the charge transfer and can dramatically increase the overpotential of the electrode. At high currents, these bubbles grow, coalesce, detach from the electrodes, leaving the floor to other new ones. Their detachments lead to voltage instabilities and oscillations^9^.

The presence of bubbles gazes in electrolyte is not inherent to metal-air batteries^10,11^. Many industrial systems related to the global context of the ecological transition face the same problems. We can cite electrolysers for alternative energy production^12^, or electrodialysis cells for desalination as examples. Electrolyte flow, specific design of the gap between the electrodes^13^ allows these technologies to prevent the apparition of large overvoltages by decreasing the ohmic resistance due to bubble coverage.

To understand and predict these effects, phenomenological models of surface coverage by bubbles for gas-evolving electrodes have been developed by Dongke Zhang and J. Eigeldinger^14–^ ^16^. Recent work from Wang^7,^^17^ includes numerical computations on bubbles formation. Using a Comsol computation and a phase field method to track the interface of the bubbles, the authors investigate the mechanisms of bubbles formation at the electrodes. They point out that coalescence of bubbles can lead to the formation of a gas film covering mainly the surface of the electrode and preventing charge transfer. No quantitative links with the electrochemical processes and their temporal evolution are displayed. Yong-liang Wang et al. proposed a numerical approach to investigate the role of gaz bubble on cell voltage oscillations. They treat an whole aluminium electrolysis cell as a resistance circuit^18^ and model the dynamic simulation of the cell equivalent circuit with Matlab/simulink software. This work shows that bubble departure frequency has an important effect on voltage oscillations. When the bubble coverage of one anode block exceeds 80 $\%$ of the surface, the cell voltage may exceed its normal fluctuation amplitude. Although interesting and meaningful, this work artificially introduces the departure frequency of the bubbles and does not relate it to either the electrochemical reaction or to the physics of the bubble detachment process.

Here, we propose numerical computations for bubble detachment on a gas evolving electrode. These computations predict the potential evolution of the electrode for a given current density while taking into account the oscillations due to local detachments. The originality of our approach lies in the description of the electrode which feature (number and heterogeneities of bubbles nucleation sites, Fritz radius associated with the site) vary as a function of the working conditions. In contrast to the previously mentioned works, we link the bubble detachment and the electrochemical reaction. This approach allows us to predict the stability of the system and the maximum current density that can be used before reaching an unstable potential. A comparison with experimental data is given. The experiments are performed on a Platinum/Carbon cell in presence or not of electrolyte flow. The first section of the article describes the principles of the simulation. We then present the experiments. Last section is devoted to the comparison between the simulations and the experimental data.

## Numerical simulation

**Figure 1:**
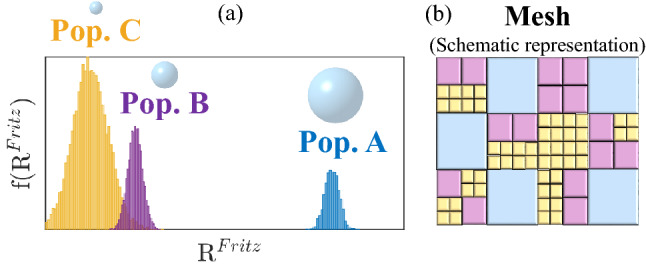
Mesh description. (**a**) Example of Fritz radii distribution showing the three populations. (**b**) Schematic example of surface meshing. Each rectangle corresponds to an element i. The size of mesh elements corresponds to its surface and corresponds to a current value given by $I(i,t)=\frac{I_{tot}S^f(i,t)}{S_{tot}}$. The color associated with each mesh element corresponds to a size of mean Fritz radius.

This section describes the simulation algorithm performed in this study. The surface of the electrode is first cut into *N* small elements. The distribution of the mesh size of the network follows a Gaussian distribution in order to reproduce inhomogeneous local dynamics. Each element is considered as an active nucleation site on which one bubble will grow. For each mesh element and for each time step we associate: the surface of the element *S*(*i*) (see Figure 1), the free surface area of the element (ie. the active surface area) where the redox reaction takes place $S^f (i,t)$, the radius of the bubble *R*(*i*, *t*) present on the surface, and a maximal radius of bubbles $R_{Fritz}(i)$ at which the bubbles detach. The Fritz radius distribution will be chosen to reflect the experimental bubble distribution. For each time step, we calculate the active surface area following Eq. (1):

1 $$\begin{aligned} S^f(i,t)=S(i)-k\pi R(i,t)^2 \end{aligned}$$

where k is assumed to be a constant taken equal to 1. We define the local current *I*(*i*) as the current passing through a small mesh element $I(i,t)=\frac{I_{tot}S^f(i,t)}{S_{tot}}$. We note $I_{tot}$ the total current and $S_{tot}$ the total surface area of the electrode. The volume of oxygen formed on each mesh element follows a Faradaic evolution :

2 $$\begin{aligned} \frac{d V_{O_{2}}(i,t)}{dt}=\frac{V_{m}I(i,t)}{nF} \end{aligned}$$

**Figure 2:**
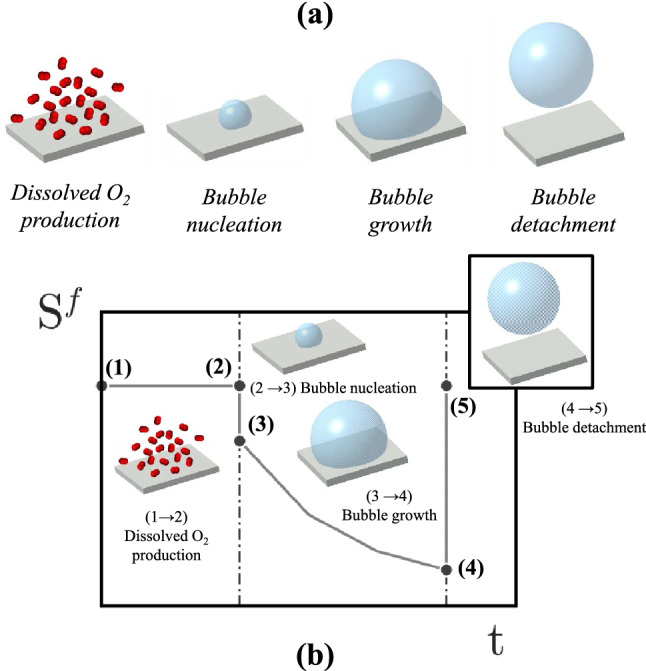
(**a**) Bubble cycle-life steps for a given mesh element. (**b**) Typical evolution of the free surface for a given mesh element. Markers 1 to 5 represents the differents steps from dissolved oxygen production to bubble detachment.

The nucleation of bubbles does not occur when the local concentration of dissolved gas reaches saturation. It is necessary to reach a high supersaturation (about 100 times greater) so that the bubble created is stable and continues to grow. When the nucleus is in such an environment, it turns into a stable bubble and its radius grows rapidly at the expense of the oxygen concentration in the electrolyte which decreases towards a value close to saturation. The volume of gas formed at each active site is then known. When this volume exceeds a critical value corresponding to the volume required to have a liquid highly saturated in oxygen above the mesh element, a bubble with a radius $R_{init}$ appears on the element (see Figure 2). We assume that $R_{init}$ does not depend upon the surface of the element and is the same on the whole electrode. The evolution of the bubble radius associated to each element follows: if $V_{O_{2}}(i,t) < V_1$

3 $$\begin{aligned} R(i,t) = 0 \end{aligned}$$

and if $$V_{O_{2}}(i,t) > V_1$$

4 $$\begin{aligned} R(i,t) = \root 3 \of {\frac{3}{4\pi }(V_{O_{2}}(i,t)-V_1)+ R_{init}^3} \end{aligned}$$

where $V_1$ is equal to $V_1=\frac{4\pi }{3}R_{init^3}+ \alpha S(i)$ with $\alpha S(i)$ the volume of oxygen dissolved at saturation in the solution other the element of size $S_i$. This crude modeling follows the studies of^19^ dealing with the formation of bubbles. The equation we propose takes this nucleation process into account but does not describe the fast growth of the nucleus radius to $R_{init}$. This phase is a short phase. The only phenomenon that is not taken into account in our study is the decrease of local conductivity of the electrolyte related to the hypersaturation phase. We neglect this mechanism with respect to the masking of the electrode by the bubble when describing the electrode potential.

A Fritz’s radius is associated with each element of the network. When the radius of the bubble reaches the Fritz’s radius $R^{Fritz}(i)$, the bubble detaches. The Fritz radius distribution follows a Gaussian distribution as described experimentally in literature^9^. Oxygen volumes and bubble radii are reset to 0 for active sites that have validated the bubble detachment test (when $R(i,t) > R_{Fritz}(i)$). A new bubble is then formed after a few time steps corresponding to the time required to saturate the solution in oxygen i.e for $V_{O_{2}}(i,t)$ to reach $V_1$. For each time step, the potential of the electrode is evaluated by the Butler-Volmer relation:

5 $$\begin{aligned} E(t)=E^0+\frac{RT}{n\alpha _0 F} \left( ln\left( \frac{S_{tot}}{\sum _{i=1}^N S^{f}(i,t)}\right) +ln\left( \frac{I_{tot}}{I_0}\right) \right) \end{aligned}$$

where $\sum _{i=1}^N S^{f}(i,t)=S^a(t)$ is the effective active surface of the electrode, $\alpha _0$, $I_0$ and $E^0$ are obtained thanks to voltamperometry experiments (see Table 1 for the nomenclature). The calculation of the potential ends the time increment considered, the algorithm performs a loop and starts again the calculations for the next time step. We anticipate that the oxygen production which is proportional to *I*(*i*, *t*) will be different at each position of the electrode because of the mesh size distribution ($I(i,t)= j S^f(i,t)$). In other words, the local growth of bubbles will be faster on some mesh elements leading to differences in bubbles sizes. Also, bubble detachment occurs at different radii because of the Gaussian distribution of $R^{Fritz}$. These differences imply the apparition of several time constants for bubbles detachment.

**Table 1:** Nomenclature.

Variable	Description	Unit (SI)
*N*	Number of mesh elements	–
$S_{tot}$	Total surface of the electrode	m$^{2}$
*S*(*i*)	Surface of element i	m$^{2}$
$\mu$	Standard deviation of mesh size distribution	–
$S^{f}(i,t)$	Free surface of element i	m$^{2}$
$S^{c}(i)$	Covered surface of element i	m$^{2}$
$S^{a}$	Total free surface of the electrode	m$^{2}$
*R*(*i*, *t*)	Bubble radius at element i	m
$R_{Fritz}(i)$	Detachment radius of bubble at the element i	m
$R_{init}$	Nucleation radius	m
*k*	Covering coefficient	–
$I_{tot}$	Total current	A
*I*(*i*, *t*)	Local current at the element i	A
$I_0$	Exchange current	A
*j*	Total current by unit surface	A.m$^{-2}$
$V_{O_2}(i,t)$	Local volume of oxygen	m$^3$
$V_{m}$	Molar volume	m$^3$ mol$^{-1}$
$V_{1}$	Nucleation volume	m$^3$
*n*	Number of electrons	–
*F*	Faraday constant	C mol$^{-1}$
*E*	Electric potential	V
$E^{0}$	Equilibrium potential	V
*R*	Universal gas constant	J mol$^{-1}$ K$^{-1}$
*T*	Temperature	K
$\alpha _{0}$	Charge transfer coefficient	–
$\sigma _{X}$	Standard deviation of Fritz radius distribution, for a population labelled X	–
$a_{X}$	Relative proportion of population labelled X	–
*f*	Probability density function of Normal law	–

### Influence of simulation parameters

As discussed in the previous section, the simulation has two Gaussian inputs: the mesh size distribution which defines the local growth dynamics; and the Fritz radius distribution which defines the local detachment size of bubbles.

We anticipate that the bubbles sizes distribution may be controlled by the design of the electrode. This justifies our analysis. We will come back to this point in the conclusion. The following paragraph is dedicated to a study of the model. We try to understand the role of the different parameters corresponding to the Gaussian distributions parameters (value of the Fritz radius, shape of the Fritz radius distribution, number of nucleation sites, and mesh size distributions). These distributions follows the probability density function of a Normal law :

6 $$\begin{aligned}&f(S)=\frac{1}{\mu \sqrt{2\pi }}e^{\frac{-1}{2}\left( \frac{S(i)-< S(i)>}{\mu }\right) ^2} \\&f(R_{Fritz})=\frac{1}{\sigma \sqrt{2\pi }}e^{\frac{-1}{2}\left( \frac{R_{Fritz}(i)-< R_{Fritz}>}{\mu }\right) ^2} \end{aligned}$$

**Figure 3:**
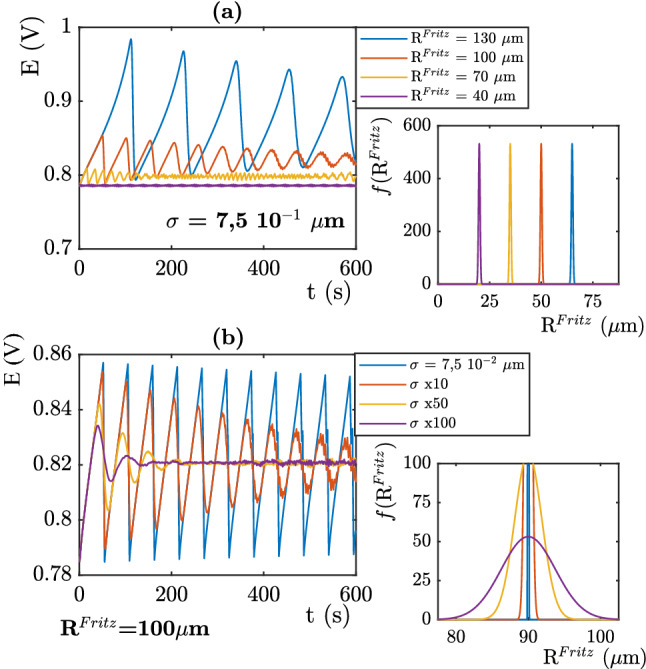
(**a**) Influence of numerical Fritz mean radius for a single population of bubbles (**b**) Influence of the numerical distribution of the Fritz radius for a single population of bubbles. j = 10 mA cm$^{-2}$, The distribution $f(R_{Fritz})$ follows a Gaussian law $f(R_{Fritz})=\frac{1}{\sigma \sqrt{2\pi }}e^{-\frac{1}{2}(\frac{R_{Fritz}-< R_{Fritz} >}{\sigma })^2}$. The distribution of the mesh size elements is given by $f(S)=\frac{1}{\mu \sqrt{2\pi }}e^{-\frac{1}{2}(\frac{S(i)-< S(i)>}{\mu })^2}$ with $<S(i)>$ = 0.015 mm $^{2}$ and $\mu$ = 5 10$^{-4}$.

   We first focus on the Fritz radius distribution : Figure 3a shows the influence of the mean Fritz radius $<R_{Fritz}>$. The signal represents the simulated electric potential for j = 10 mA cm$^{-2}$. These calculations are performed for a given mesh element size distribution: $<S(i)>$ = 0.015 mm $^{2}$ and $\mu$ = 5 10$^{-4}$. This highly centered mesh size distribution allow to get rid of the influence of inhomogeneous current repartition on the calculated signal.

The signal represented on Fig. 3 corresponds to several mean value of $<R_{Fritz}>$. Low $<R_{Fritz}>$ values correspond to low overpotential, oscillations with low amplitude and high frequency. These behavior are easily explained. From the onset of the current, oxygen is produced at the nucleation sites, and generates the creation of bubbles that cover the surface. For a peaked distribution with a low standard deviation the detachment of bubbles at the different sites are in phase. In this situation, the effective surface of the electrode oscillates between $S_{tot}$ and $S_{tot}-N k\pi <R_{Fritz}>^2$ as a function of time where *N* is the number of sites and is given by $N=\frac{S_{tot}}{<S(i)>}$. We have here considered that for a peaked distribution $<R_{Fritz}>^2=<R_{Fritz}^{2}>$. For low Fritz radius, the bubbles detach quickly from the electrode because they reach $R_{Fritz}$ faster, which then generates fast detachment frequencies. Thus, decreasing the Fritz radius reduces the covered area and increases the efficiency of the process: the measured and its oscillations amplitudes are lower.

The influence of the standard deviation $\sigma$ is displayed on Fig. 3b, using a mean value $< \hbox {R}_{Fritz} > = 100$ $\mu$m. The signals reach the same average value, however, oscillations amplitudes decrease while $\sigma$ increases. When $\sigma$ vanishes, the bubbles grow, cover the electrode surface and finally detach suddenly at the same radius and thus at the same time. It results brutal drops of potential induced by this cohesive movement. Conversely, an increase in $\sigma$ corresponds to a wider distribution of the Fritz radius. Highly heterogeneous distributions will give a wide distribution of detachment time at each element. Indeed, because of this wide distribution, some bubbles are still growing while others have already left the surface. This mechanism induces a decrease of the oscillations amplitude, the signal finally reaches a stable regime with a steady value.

**Figure 4:**
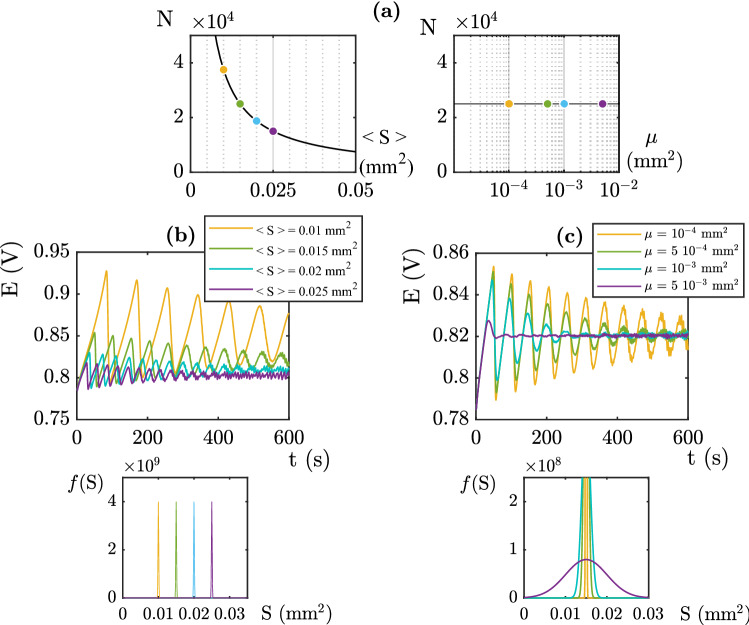
(**a**) Relation between mesh distribution parameters and number of elements. (**b**) Influence of the mean mesh size on a single population of bubbles. (**c**) Influence of the mesh size elements distribution on a single population of bubbles.j = 10 mA cm$^{-2}$.The Fritz radius distribution is given by, $<R_{Fritz}>$ = 100 $\mu$m. $\sigma$ = 7.5 10$^{-4}$.

The second input to the simulation corresponds to the mesh size distribution of the network. This distribution defines the local growth dynamics since $I(i,t) = j S^f(i,t)$. but also the number of bubbles nucleation sites, $N=\frac{S_{tot}}{< S(i) >}$. The evolution of *N* with the mesh distribution parameters is represented in Fig. 4a. In this part of the study, the Fritz radius distribution is given by $<R_{Fritz}>$ = 100 $\mu$m, $\sigma$ = 7.5 10$^{-4}$. Fig. 4b displays the role of $< S(i)>$ on the electro-chemical signals. We observe a lower electric overpotential for high mean surfaces. Increasing $< S(i)>$ i.e decreasing *N* reduces the amplitudes of the oscillations and gives a faster dynamics of the detachment which, leads to a low and stable electric potential. We note that the average electrical potential increases as a function of *N*. As previously, this behavior comes from the temporal evolution of the covered surface. The covered surface per site is given by $k\pi R(i,t)^2$ where *R*(*i*, *t*) is given by Eqs. (3), (4). Thus as a function of time the effective surface of the electrodes oscillates between $S_{tot}$ and $S_{tot}-k\pi N <R_{Fritz}>^2$. Following Eq. (5), this causes the overpotential to increase as $ln\left( \frac{S_{tot}}{S_{tot}-k\pi N <R_{Fritz}>)^2}\right)$. The time dependence of the electrical signal is hidden in the growth of the bubble radius. When $\mu =0$ is, the behavior of each bubble is the same and the period of the signal is given by $T=\frac{N(n F(R_{Fritz}^3-R_{init}^3)+V_1)}{V_m I_{tot}}$. Thus the measured oscillations have a proportional period which increases as *N*. A larger mean surface leads to smaller overpotentials and shorter temporal oscillations. This analysis corresponds to a situation where the size distribution is very tight with a low standard deviation as presented in Fig. 4b.

**Figure 5:**
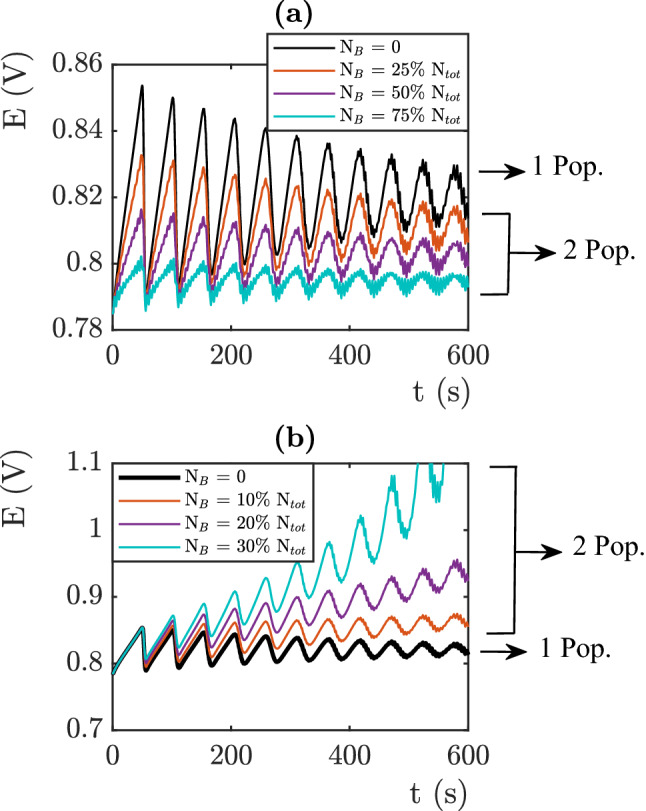
(**a**) Influence of a second population of $R^{Fritz}$ = 45 $\mu$m on the electric potential (**b**) Influence of a second population of $R^{Fritz}$ = 400 $\mu$m on the electric potential. j = 10 mA cm$^{-2}$ .

Figure 4c shows the influence of the distribution of the size of the mesh elements. The observed behavior is similar to the previous analysis in Fig. 3b: for wide distributions, steady state regimes are obtained in shorter times. Indeed, when the size of nucleation sites (ie. mesh elements) are heterogeneous, current distribution is heterogeneous and phases-shift occurs rapidly. In this case, some bubbles display a faster growth and reach the Fritz radius, while other bubbles are still slowly growing at the surface. This common behavior between Fig. 4c and Fig. 3b is due to the same phenomena (phases-shift) but has different origin. The Fritz radius distribution (Figure 3b) corresponds to heterogeneities in terms of detachment radius, while the mesh elements distribution (Figure 4c) corresponds to heterogeneous kinetics. In both cases, a broad distribution leads to a more stable behavior.

To further analyze the influence of the parameters, we will now study the impact of the presence of multiple bubble populations. Figure 5a shows the signal calculated when a second population with a smaller Fritz radius is added to a first population whose Fritz radius was fixed at 100 $\mu$m. The mesh size distribution is set to $f(S(i))=\frac{1}{\mu \sqrt{2\pi }}e^{-\frac{1}{2}(\frac{S(i)-< S(i)>}{\mu })^2}$ with $<S(i)>$ = 0.015 mm $^{2}$ and $\mu$ = 5 10$^{-4}$.

The black line represents the signal for a single population. The colored graphs show the influence of the second population in several proportions. The number of nucleation sites *N* is fixed in these comparisons and the mesh size follows the same Gaussian distribution for each plots. The behavior of the small bubbles is different from that of the large ones. The small bubbles will cover a smaller area and will stay on the electrode for shorter times. This has two consequences. The decrease of the covered surface leads to a decrease of the average value of the overvoltage. The faster departure of the small bubbles from the electrodes causes a high frequency component to appear in the electrical signal. Figure 5b shows what happens in the presence of a population with a higher Fritz radius. The large bubbles do not reach their critical radius during the time of the experiment. Therefore, they lead to a constant increase of the overvoltage. This last analysis shows the importance of each population on the electric signal. Small Fritz radii induce high frequency oscillations while high ones induce high times constant during which the overpotential increases constantly. We understand here that it is necessary to have several Fritz radius distribution to obtain a signal with several time constants. The next section provides a comparison of simulation and experimental datas, we will see that the experimental measured signals have dynamics governed by several time constants. This behavior is then consistent with visual observations and the multi-modal simulation results presented here.

### Modeling: choice of the mesh size and of the Fritz radius parameters

In this section, our goal is to verify that the model described above can account for the experimental data we have measured. To do this it is necessary to define the distribution of nucleation sites and the distribution of Fritz radii. As discussed previously, the simulation inputs are Gaussian vectors which translate the physical reality of the system. We define a Gaussian distribution for the surface of the mesh elements. This distribution is centered on a value $< S(i)>$ and has a standard deviation of $\mu$. We choose N elements that fill this distribution and multiply their size by a factor $\beta$ so that the total area of the N elements corresponds to the total area of the electrode. Each element is then associated with a Fritz radius $R^{Fritz}(i)$. The experimentally observed distribution of the bubbles (^9,20^) is introduced inside the simulation by the separation of active sites into three types. In this work, we decide to work with three population of bubbles. The first type corresponds to active sites giving large bubbles (population A), the other sites produce bubbles of smaller size (population B) and the latest produce bubbles of the smallest size (population C). These considerations are based on experimental data^9,20^. In the low current density situation, the distribution of bubbles is wide, centered on a well defined radius. In the high current density situation, a second peak appears in the bubble size distribution, which is much smaller in size than the previous ones^9,20^. This peak is interpreted by considering that the action of the current on the metal surface creates modifications of the oxide layers which can allow the appearance of other types of nucleation sites. Let us note that it is necessary to call upon several types of nucleation site to have bubbles of different size. Experiments performed on an electrode with designed cavities gives a single mode distribution^21^. A population of large bubbles in a low number can also be observed. We did not report the feature of this population of bubbles in our previous studies because their small number made our statistics very poor^9^. The introduction of the large bubbles is necessary here to account for the temporal low frequency oscillations of the electric potential. These large bubbles may be produced by coalescence events. Following these considerations, we thus propose to use a tri-modal description where each mode follows a Gaussian law. At the beginning of the simulation we decide the value of the Fritz’s radius for each site and make this association through a random draw. We anticipate that due to the high number of mesh elements and to the low standard deviation of the various size distributions, the calculated electrical potential will not depend upon the association of a site of size *S*(*i*) and of one Fritz radius $R_{Fritz}(i)$ but only from the two distributions. In other words two successive random draw leads to two different associations, but to potential signal evolution which are so closed that we are not able to distinguish them through a mean square analysis.

To summarize, our model contains 10 fitting parameters: *N* the numbers of elements of the mesh, $\mu$ the standard deviation of the elements area distribution, the mean Fritz radius for the A (respectively B,C) population R$_A$ $_{Fritz}$, (respectively R$_B$ $_{Fritz}$, and R$_C$ $_{Fritz}$,), the standard deviation of these three distributions $\sigma _A$, $\sigma _B$, $\sigma _C$ and the relative importance of the distribution $a_A, a_B$ with $a_A=\int _{A population} f(x)dx$,$a_B=\int _{B population} f(x)dx$,$a_C=\int _{C population} f(x)dx$, the integrals are calculated on the populations A B C respectively, f is the probability density of the size distribution. $a_C=\int _{C population} f(x)dx$ is not a fitting parameter, and is given by $a_A+a_B+a_C=1$.

### Overvoltage prediction

**Figure 6:**
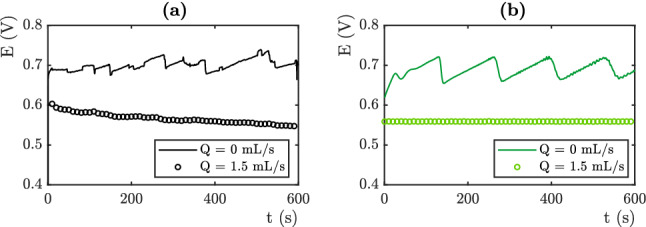
(**a**) Electrical Potential versus Ag/AgCl electrode as a function of time, Experimental (**b**) Simulation. j = 3 mA cm$^{-2}$.

**Figure 7:**
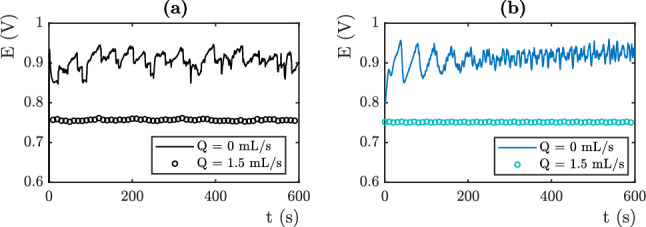
(**a**) Electrical Potential versus Ag/AgCl electrode as a function of time, Experiments. (**b**) Simulations. j = 10 mA cm$^{-2}$.

**Figure 8:**
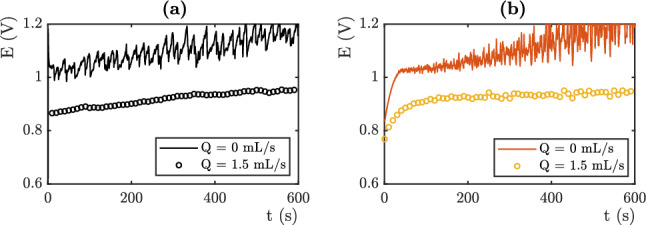
(**a**) Electrical Potential versus Ag/AgCl electrode as a function of time, Experimental (**b**) Simulation.j = 20 mA cm$^{-2}$.

The numerical simulation results are compared to the experimental data obtained from the device presented in the experimental section. Interestingly, these experiments concern very different study cases. Changing the value of the electrolyte flux allows the Fritz radius to be varied and thus generates data where these differ. The electrical potential of the OER platinum electrode is measured against an Ag/AgCl reference electrode (saturated with KCl). Figure 6 (respectively Figures 7, 8) displays the results obtained for j = 3 mA cm$^{-2}$, respectively j = 10 mA cm$^{-2}$ and j = 20 mA cm$^{-2}$. The black lines represent the experimental results (see Figures 6a , 7a and 8a), while the colored lines represent the computations (see Figures 6b, 7b and 8b). To capture this behavior, we use the Fritz radius distribution function and the mesh element size distribution displayed in Tables 2 and 3.

The numerical computations predict electrical potentials values which are in agreement with the experiments. The values of the 10 fitting parameters determine the values of the mean measured overpotential. These latter is highly sensitive to the variation of each fitting parameters. The experiments and the simulations may show differences in the early stages. This is due to the experimental difficulty to control the initial conditions and the presence of residual bubbles on the electrode. We set the values of the fitting parameters on the last 300 seconds.

In the absence of flow, the measured electrical potentials are higher than in the presence of flow. This is related to the coverage of the electrode surface by oxygen bubbles. At low current densities (j = 3 mA cm$^{-2}$), according to the Butler-Volmer equation the theoretical potential is equal to 0.55 V vs Ag/AgCl. The potential measured in flow reaches 0.55 V vs Ag/AgCl. The overvoltage due to the presence of bubbles on the surface is then 0 mV. On the other hand, it reach 100 mV in static electrolyte conditions. By increasing the current density, the overvoltage related to the bubbles using flowing electrolyte increases. At high current densities (j = 20 mA cm$^{-2}$), total evacuation of the bubbles at the surface of the electrode should theoretically (according to Butler-Volmer relation) give a potential around 0.75 V (vs Ag/AgCl). The value measured in our system with flowing conditions is 0.95 V (vs Ag/AgCl) and oscillates between 1.05 and 1.2 V using static electrolyte. The value of the overpotential is mainly given by the number of nucleation sites which fixes the proportion of the covered surface. The mean surface of the mesh element decreases from 0.03 mm$^2$ for j=3 mA cm$^{-2}$, to 0.015 mm$^2$ for j=10 mA cm$^{-2}$ and 0.009 mm$^2$ for j=20 mA cm$^{-2}$. This variation captures the increase of the overpotential.

Note that the distribution of Fritz radii, at least the average values of the radii of the three modes, does not vary much. The Fritz radius of the largest population even decreases as a function of current density which does not favor the increase of the overpotential. We therefore conclude that the value of the overpotential is mainly governed by the number of nucleation sites in this situation. The Fritz radius distribution is at the origin of the temporal dynamics.

**Table 2:** Simulation parameters used for static electrolyte experiments.

Current	**Mesh**		**Pop.A**		**Pop.B**		**Pop.C**		**Repartition**		
j (mA cm$^{-2}$)	N	$\mu$ ($\mu$m$^2$)	R$_A$ $_{Fritz}$ ($\mu$m)	$\sigma$ $_A$	R$_B$ $_{Fritz}$ ($\mu$m)	$\sigma$ $_B$	R$_C$ $_{Fritz}$ ($\mu$m)	$\sigma$ $_C$	^a^$_A$	^a^$_B$	^a^$_C$
3	12500	5	400	10	120	0.75	75	5	1$\%$	25$\%$	74$\%$
10	25000	5	400	10	95	1.5	62.5	5	1$\%$	25$\%$	74$\%$
20	41667	5	250	10	80	10	43	40	1$\%$	25$\%$	74$\%$

**Table 3:** Simulation parameters used for flowing electrolyte experiments (Q = 1.5 mL/s).

Current	**Mesh**		Pop.A		Pop.B		Pop.C		Repartition		
j (mA cm$^{-2}$)	N	$\mu$ ($\mu$m$^2$)	R$_A$ $_{Fritz}$ ($\mu$m)	$\sigma$ $_A$	R$_B$ $_{Fritz}$ ($\mu$m)	$\sigma$ $_B$	R$_C$ $_{Fritz}$ ($\mu$m)	$\sigma$ $_C$	^a^$_A$	^a^$_B$	^a^$_C$
3	12500	5	30	10	30	0.5	30	5	1$\%$	25$\%$	74$\%$
10	25000	5	30	10	30	0.5	30	5	1$\%$	25$\%$	74$\%$
20	41667	5	30	10	30	60	30	60	1$\%$	25$\%$	74$\%$

In the absence of flow, the electric potential varies with time and shows oscillations. The oscillations observed are associated to bubbles detachments, which cause variations of the active surface of the electrode. Their frequency and their amplitude depend on the departure of the bubbles. Describing the temporal evolution is achieved by using bubbles of different sizes. At a given masked surface, the amplitude of the oscillations is controlled by the width of the mesh size distribution. A very small width would lead to large oscillations and a periodic signal. Indeed, bubbles that all have the same radius will evolve in a similar way and detach at the same time. The masking of the surface will be very important or very weak depending upon the time, which will cause important variations of surface and electrical potential. The Fritz radius distribution are given in Table III. These distributions are in agreement with the one measured in^9^. They are roughly the same for j=3 mA cm$^{-2}$ and j=10 mA cm$^{-2}$. The size of the medium population decreases as a function of the current density. Let us recall that the main consequence of enhancing the density current is to increase the number of nucleation site and that this parameter is of first importance to set the overpotential . The amplitude of oscillations are roughly the same in both situations (50 mV). The oscillations display shorter time scales because the time required to fill a bubble is shorter when the density current is higher. Even though the mesh element surface are two times smaller, the local density currents are higher since the total intensity is three time higher. They are different for j=20 mA cm$^{-2}$. The distribution of the smallest bubble is wide which induces a noisy signal with a small amplitude.The largest peak are due to the departure of the largest bubbles which are not numerous. The experimental signal seems more periodic than the numerical one. This may come from coalescence events that are not taken into account in the numerical estimations.

In the presence of flow, the electrical overpotential is lower and we note no temporal fluctuations. This is captured in the numerical simulation, by using a wide single population of small bubbles. Big bubbles are evacuated by the flow. The width of the single population of small bubbles allows to avoid oscillations in the electrical potential signal. Bubbles leave the surface at different times and an average surface is always covered.

## Conclusion

In this work we have presented a numerical simulation to predict the evolution of the electrical potential of a battery or electrolyser when the oxidation-reduction reactions involve a gas. The simulation allows to find the experimental results in a convincing way. It allows to predict the value of the potentials as well at the equilibrium as during the transient stage as well as the oscillations of this potential. The fitting parameters used are close to the one measured^9^.

This work allows us to give advice for the manufacture of electrodes. To avoid too high overpotentials, it is necessary to reduce the masking of the cell. Producing a large number of small bubbles that escape quickly allows to have a lower average masking and thus a lower overpotential. As shown in the numerical simulations, a population of small bubbles with a large standard deviation allows to limit the overpotential but also to avoid signal oscillations.

To reach this aim, it is necessary to be able to control the bubble size distribution. One way to do this is to do this is to create artificial sites of preferential nucleation on the electrode that set different Fritz radii. Brussieux et all showed^22^ that this was possible by creating hydrophobic spots on electrodes. All techniques that allow to vary the wettability of the electrode (by oxidation) or that modify the geometry of the surface (cavities) may be used. Note that the creation of artificial nucleation site is also an approach that allows in the context of porous electrodes to ensure that bubbles do not form in the pores. This limits the mechanical degradation of the porous electrode and facilitates their removal^19^. This work opens the door to the design of new performant electrodes.

To conclude, we would like to clarify one last point. We have insisted a lot on the harmful character of the formation of bubbles on the value of the potential. The decrease of the contact area of the electrode is clearly a prohibitive point. It is obvious that it is impossible to use a gas electrode without making bubbles. Our advice here is to create bubbles of small radius. Our study shows that excessively small bubbles would be the ideal solution to optimize the performance of gas electrodes. The value of this radius must be fixed also taking into account the fact that a gas-liquid interface can have a positive effect and catalyze a reaction^23^. The right critical radius must therefore be as small as possible to prevent the anchoring of bubbles but also allow these catalytic effects. An optimal size may have to be chosen. Incorporating catalytic effects into the model and finding this equilibrium size is one of the perspectives of this work.

## Experimental section

The data that will be used for the comparison with the modeling have been previously reported in^9^. We describe here briefly the main steps of the experimental procedure. Details can be found in^9^.

### Electrodes and electrolyte

The studied gas evolving electrode is a platinum plate (2,5 x 1,5 cm, thickness = 0.25 mm) from Sigma-Aldrich, the counter-electrode is a carbon plate with the same dimensions.An Ag/AgCl electrode is inserted in the cell and will be used as a reference electrode. The electrolyte is an 8M potassium hydroxide aqueous solution obtained by dissolving KOH solid pellets in distilled water. These conditions are similar to those of electrolyzers and metal-air cells. The platinum electrode is a catalyst for the OER reaction and allows also to study and simulate the effect of the flow without significant side reactions. This makes platinum electrode as an efficient model electrode for studying the gas evolution process at its surface.

### Electrochemical flow cell

**Figure 9:**
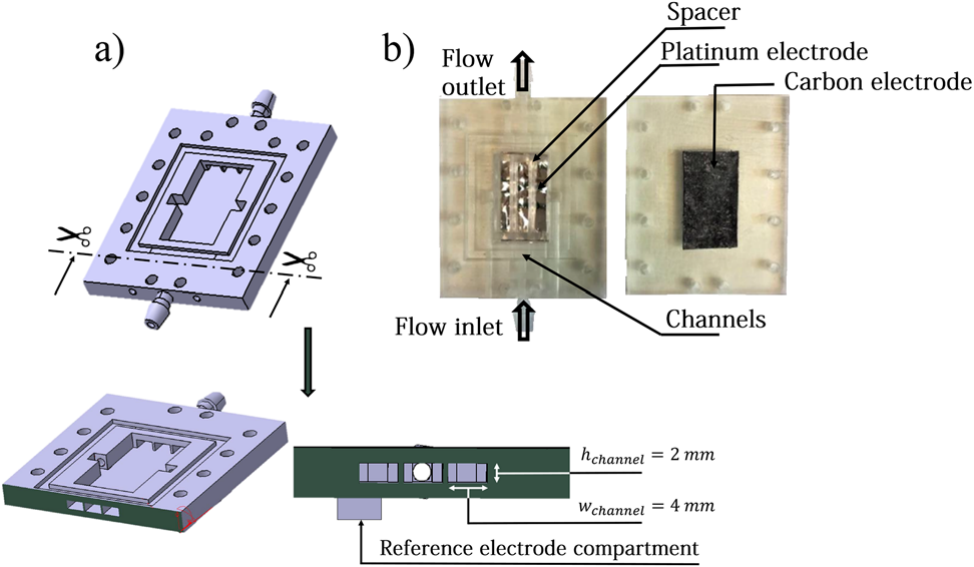
(**a**) CAD design of the flow cell, general view and cross-sectional view. (**b**) Picture of the cell.

The electrochemical flow-cell is 3D printed using stereolithography process. The CAD design is presented in Fig. 9. Electrodes are hold as shown in Fig. 9, a spacer sets the distance between the electrodes at 2 mm.

The electrolyte is injected inside the inlet channels, then flows through the cell and comes out along the outlet channels. A peristaltic pump is used to impose the flow rate of the electrolyte. The Reynolds number is given by :

7 $$\begin{aligned} Re = \frac{Q \rho }{\eta S}L_c \end{aligned}$$

This geometry leads to laminar flow with Re comprised between 0 and 600 for flow rates up to 1,5 mL/min.

### Tafel measurements

**Figure 10:**
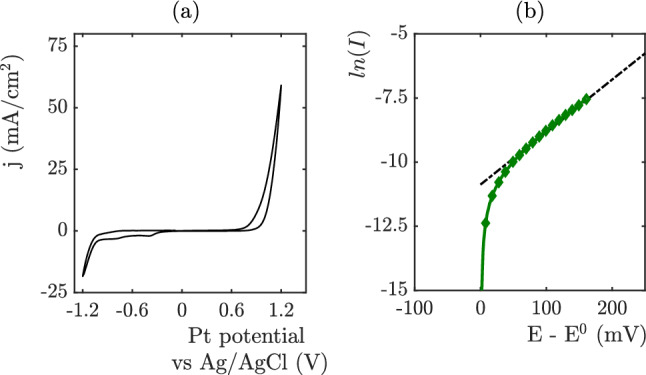
(**a**) Cyclic voltammetry measurements are performed on the OER platinium electrode at a scan rate of 50 mV/s. (**b**) Tafel plot.

Cyclic voltammetry measurements are performed on the OER platinum electrode at a scan rate of 50 mV/s. An Ag/AgCl reference electrode (KCl sat.) is used for the working electrode potential.The results of cyclic voltammetries are shown in Fig. 10. These experiments are performed without electrolyte flow. The oxidation reaction starts around 0.55 V vs Ag/AgCl. This potential corresponds to hydroxide ion adsorption and fast oxidation of platinum. These reactions are directly followed by oxygen production^24^ at higher voltage. At ph=13.9, Favaro et all^24^ show that 30$\%$ of the current corresponds to surface oxydation and 70$\%$ of the current corresponds to OER at j=1 mA cm$^{-2}$. The proportion of current coming from surface oxydation vanishes at higher current. In our work, we need to model the density current for j higher than 3 mA cm$^{-2}$. In this situtation, the current follows the Tafel law: $I=I_o.exp^{\frac{\alpha _O F(E-E^O)}{RT}}$. The curve fitting leads to $E^0$ = 0.55 V , $I_o$=1.03 $10^{-3}$ A, and $\alpha _O=0.06$, $E^0$ = 0.55 V.

### Data acquisition

Chronopotentiometry experiments are performed to investigate and reproduce by simulation the effect of bubbles detachment at the platinum gas evolving electrode. All the experiments are performed using a potentiostat (Bio-Logic, VSP200). A constant current is applied, the electric potential of the platiumum electrode versus a Ag/AgCl reference electrode is recorded over time. Before applying the current density, a strong flow (Q= 10ml/min) is applied in the cell for 5 minutes in order to sweep the residual bubbles.The flow is then stopped after one minute the electrical current is applied.

## Data Availability

The datasets used and/or analysed during the current study available from the corresponding author on reasonable request.
